# Prevalence of myopia among senior students in Fenghua, Eastern China, before and during the COVID-19 pandemic

**DOI:** 10.3389/fpubh.2023.1180800

**Published:** 2023-06-06

**Authors:** Xuewei Zhou, Tong Liu, Aimin Wu, Bo Cheng, Min Chen, Kaijun Wang

**Affiliations:** ^1^Department of Ophthalmology, The People’s Hospital of Fenghua, Ningbo, Zhejiang, China; ^2^Eye Center, The 2nd Affiliated Hospital, Medical College of Zhejiang University, Hangzhou, China; ^3^Zhejiang Provincial Key Laboratory of Ophthalmology, Hangzhou, China

**Keywords:** myopia, prevalence, senior students, COVID-19, academic stress

## Abstract

**Background:**

Myopia is a common cause of vision impairment worldwide. In China, the prevalence, the affected population, and the onset age of myopia are prominent issues. Prevention and intervention of myopia are great public health concerns.

**Methods:**

This school-based retrospective study retrieved visual acuity and refractive data of senior students (grade 12th) from six high schools in Fenghua City, Zhejiang Province, eastern China, from 2016 and 2022. Noncycloplegic autorefraction was performed for refractive status. Students were divided into three subgroups by their school types. The overall myopia prevalence, as well as the prevalence of low myopia, moderate myopia, and high myopia, were calculated separately for each year. Statistical analyzes were performed using SPSS 25.0 and Graphpad Prism software.

**Results:**

The mean myopia prevalence in Fenghua was 84.5% (95% CI: 84.0–85.0%), and a slightly downward trend was found in myopia prevalence after 2019, but the change was not statistically significant (*p* = 0.078). The overall prevalence of myopia was 79.6, 85.2, and 86.1% in vocational high schools, general high schools, and key high schools, respectively, with statistically significant differences (*p* < 0.001). The prevalence of myopia among senior students in the vocational high school was significantly lower than that in the other two high schools. There’s no significant change in the overall prevalence of myopia (84.7% vs. 84.3%, *p* = 0.265) before and during the COVID-19 pandemic, and it remained statistically insignificant after stratifying by gender (male *p* = 0.207, female *p* = 0.918) or school types (vocational high school *p* = 0.112; general high school *p* = 0.299; key high school *p* = 0.393).

**Conclusion:**

The prevalence of myopia among senior students in Fenghua is relatively high, and the COVID-19 pandemic has no significant impact on it. The prevalence of myopia among vocational high school students is lower than that of general high school and key high school. Attention should be paid to the effects of educational pressure on the prevalence of myopia among students.

## 1. Introduction

Myopia is a common cause of vision impairment worldwide. It is now widely acknowledged that high myopia increases the risk of ophthalmic pathological changes, such as retinal detachment, myopic macular degeneration, glaucoma, and cataracts (1–3), which may eventually result in irreversible vision impairment (4). Myopia, especially high myopia, has a negative impact on public health and quality of life, and it leads to significant healthcare costs and productivity losses (5, 6).

Asia has long been considered to have a high prevalence of myopia (6–8), particularly in China, issues such as the high prevalence of myopia and its fast growth are prominent (5, 7, 9). In our previous study, we have found that from 2001 to 2015, the prevalence of myopia among high school students in Fenghua increased from 79.5 to 87.7%, with a notable increase in the prevalence of high myopia (10). Moreover, changes in myopia prevalence during the COVID-19 pandemic are also issues of interest, with a number of studies reporting an increase trend in myopia prevalence during this period (11–13). For instance, a study conducted in Chongqing, China found that the prevalence of myopia rose from 45.3 to 55.4% following the COVID-19 outbreak (14), and another study also revealed an increase of myopia prevalence by 5.8% compared to 2019 (11).

In the current study, we evaluated changes of myopia prevalence among senior students from six high schools in Fenghua, from 2016 to 2022. We also compared the time trend of myopia prevalence before and during the COVID-19 pandemic aiming to provide some valuable information for myopia prevention and control in China.

## 2. Methods

### 2.1. Study population

This school-based retrospective study retrieved visual acuity and refractive data of senior students (grade 12) from six high schools (including 1 vocational high school, 4 general high schools, and 1 key high school) in Fenghua City, Zhejiang Province, eastern China, from 2016 and 2022. Senior students were required to receive a physical checkup before the college entrance examination each year. All the ophthalmic examinations were routinely conducted by experienced ophthalmologists and optometrists from Fenghua People’s Hospital, and the results were recorded in the database. Ethical approval was obtained from the ethics committee of Fenghua People’s Hospital. The study adhered to the tenets of the Declaration of Helsinki.

### 2.2. Ophthalmic examination

All students underwent a Standard Logarithmic Visual Acuity E chart test at 5-meter for uncorrected visual acuity (UCVA). If UCVA was less than 5.0, best corrected visual acuity (BCVA) was measured with subjective refraction. Noncycloplegic autorefraction was performed by autorefractor (AR-600; Nidek Ltd., Tokyo, Japan), and the spherical equivalent refraction (SER) was calculated as the spherical refraction plus half of the cylindrical refraction. A slit lamp examination was performed to exclude opacity of the optic media. A self-reported information about previous ophthalmic medical history was also recorded for analysis, including ophthalmic disease, wearing contact lenses or orthokeratology, and/or refractive surgery.

### 2.3. Definition of refractive status

Due to a good correlation between the right and left eyes (*r* = 0.912, *p* < 0.001; Table 1), the SER of the right eye from each student was chosen for analysis, which was defined as following: non-myopia (SER > −0.5 D), low myopia (−3.0 D < SER ≤ −0.5 D), moderate myopia (−6.0 D < SER ≤ −3.0 D), and high myopia (SER ≤ −6.0 D).

**Table 1:** Correlation of SER between right and left eyes.

Year	Right median (IQR)	Left median (IQR)	*P* value^a^	*r*
2016	−3.5(3)	−3.0(4)	<0.001	0.920
2017	−3.5(3)	−3.0(3)	<0.001	0.919
2018	−3.5(4)	−3.0(3)	<0.001	0.923
2019	−3.5(3)	−3.5(4)	<0.001	0.919
2020	−3.5(3)	−3.5(4)	<0.001	0.908
2021	−3.5(3)	−3.0(4)	<0.001	0.902
2022	−3.0(3)	−3.0(4)	<0.001	0.888
Total	−3.5(3)	−3.0(4)	<0.001	0.912

SER, spherical equivalent refraction; IQR, interquartile range. ^a^Spearman correlation.

### 2.4. Statistical analysis

Skewed distribution data was described as medians (interquartile range, IQR), and percentiles were used to describe categorical variables. The overall myopia prevalence, as well as the prevalence of low myopia, moderate myopia, and high myopia, were calculated separately for each year. Changes and differences in myopia prevalence between genders and high schools were analyzed using chi-square tests for dichotomous data or rank sum tests for ranked ordinal data. Correlations of SER between left and right eyes were assessed using the Spearman rank correlation test. Statistical analyzes were performed using SPSS 25.0 (SPSS Inc., Chicago, Illinois, United States) and Graphpad Prism software version 8.0 (Graphpad software Inc., SanDiego, CA, United States) unless otherwise noted, and a *p* value less than 0.05 was considered statistically significant.

## 3. Results

### 3.1. General characteristics

Basic characteristics of the study population were summarized in Table 2. Totally, 17,304 senior students from six high schools in Fenghua City were included in the study from 2016 to 2022. Among them, 118 students were excluded due to history of refractive surgery (*n* = 49), orthokeratology (*n* = 43), hyperopia (*n* = 4), other ophthalmic diseases (*n* = 18), and data missing (*n* = 4). Finally, 17,186 students were included for analysis, consisting of 8,107 males (47.1%) and 9,079 females (52.8%), and the ratio of gender was shown in Table 2. Overall, there was no significant difference in gender ratio among the three high schools (*p* > 0.05, except for 2016 *p* < 0.001).

**Table 2:** Basic characteristics of the study population.

Year	*N*	Male/Female	Vocational high school (Male/Female)	General high school (Male/Female)	Key high school (Male/Female)	*P* value^a^
2016	2,718	1294/1424	361 (135/226)	1874 (936/938)	483 (223/260)	<0.001
2017	2,468	1178/1290	287 (131/156)	1707 (834/873)	474 (213/261)	0.240
2018	2,442	1174/1268	234 (122/112)	1760 (831/929)	448 (221/227)	0.309
2019	2,360	1091/1269	407 (185/222)	1,482 (694/788)	471 (212/259)	0.743
2020	2,332	1090/1242	365 (169/196)	1,504 (724/780)	463 (197/266)	0.107
2021	2,354	1089/1265	447 (207/240)	1,451 (658/793)	456 (224/232)	0.370
2022	2,512	1191/1321	527 (256/271)	1,558 (713/845)	427 (222/205)	0.062
Total	17,186	8107/9079	2,628	11,336	3,222	0.279

^a^Comparison of male/female ratio between different types of schools, Chi-square test.

### 3.2. Uncorrected visual and refractive status

**Figure 1:**
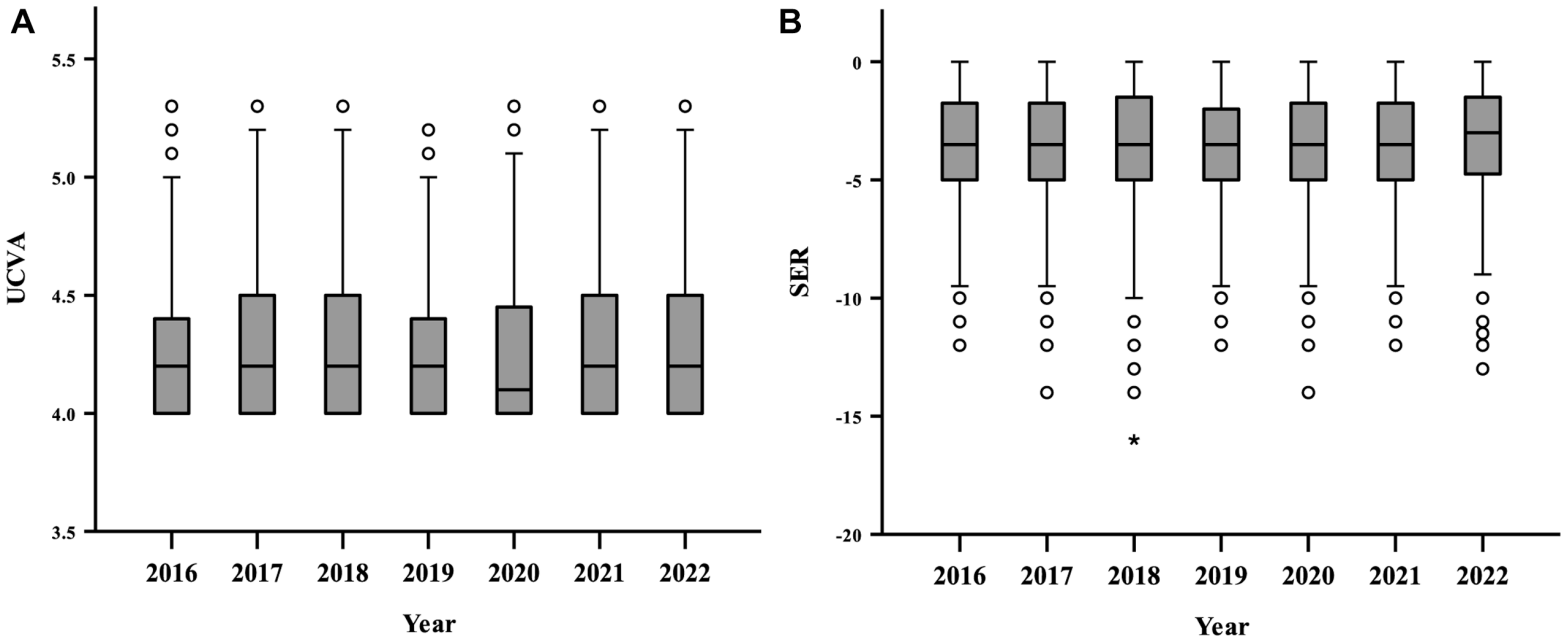
The refractive status of senior students in Fenghua from 2016 to 2022. **(A)** Uncorrected visual acuity (UCVA); **(B)** Spherical equivalent refraction (SER). * and ° represent outlier data: °greater than Q3 + 1.5IQR or less than Q1-1.5IQR, *less than Q1-3IQR. Q, quartile; IQR, interquartile range.

Figure 1 and Table 3 showed the mean UCVA and SER of senior students in Fenghua from 2016 to 2022 that both of them were fluctuating (UCVA *p* < 0.001, SER *p* < 0.001), but not obviously along the time trend.

**Table 3:** The mean uncorrected visual acuity and refractive status of senior students in Fenghua, from 2016 to 2022.

	UCVA Median (IQR)	SER Median (IQR)	Myopia prevalence (%)				
			Total prevalence	Male (95%CI)	Female (95%CI)	OR (95% CI)	*P* value^a^
2016	4.2 (0.4)	−3.5 (3)	85.6 (84.2–86.9)	83.2 (81.0–85.2)	87.9 (86.0–89.5)	1.465 (1.181–1.817)	<0.001
2017	4.2 (0.5)	−3.5 (3)	84.2 (82.7–85.7)	81.2 (78.8–83.4)	87.1 (85.1–88.8)	1.562 (1.255–1.943)	<0.001
2018	4.2 (0.5)	−3.5 (4)	83.3 (81.8–84.8)	80.9 (78.6–83.1)	85.5 (83.4–87.4)	1.389 (1.122–1.720)	0.002
2019	4.2 (0.4)	−3.5 (3)	85.6 (84.1–86.9)	83.7 (81.4–85.8)	87.2 (85.2–88.9)	1.323 (1.051–1.665)	0.017
2020	4.1 (0.5)	−3.5 (3)	85.2 (83.7–86.7)	82.6 (80.2–84.8)	87.6 (85.6–89.4)	1.491 (1.185–1.877)	0.001
2021	4.2 (0.5)	−3.5 (3)	84.3 (82.7–85.7)	80.3 (77.8–82.6)	87.7 (85.8–89.5)	1.762 (1.407–2.206)	<0.001
2022	4.2 (0.5)	−3.0 (3)	83.3 (81.8–84.7)	80.6 (78.2–82.8)	85.7 (83.7–87.5)	1.441 (1.168–1.779)	0.001
*P* value	<0.001^b^	<0.001^b^	0.078^a^	0.208^a^	0.372^a^		

UCVA, uncorrected visual acuity; SER, spherical equivalent refraction; OR, odds ratio; CI, confidence interval. ^a^Chi-square test, female vs male; ^b^Mann–Whitney *U* test.

### 3.3. Prevalence of myopia in different genders

**Figure 2:**
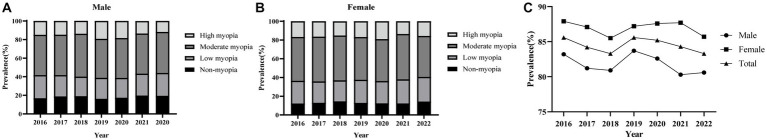
Prevalence of myopia by gender. **(A)** The distribution of different degrees of myopia prevalence in males; **(B)** The distribution of different degrees of myopia prevalence in females; **(C)** Trends in myopia prevalence.

The mean myopia prevalence in this region was 84.5% (95% CI: 84.0–85.0%). A slightly downward trend was found in myopia prevalence after 2019 in Figure 2, but the change was not statistically significant (*p* = 0.078). Table 3 shows the overall prevalence of myopia was consistently higher among females than males (OR = 1.485, 95% CI: 1.367–1.614, *p* < 0.001). In terms of the prevalence of different degrees of myopia, the distribution of prevalence among males changed in low myopia, moderate myopia, and high myopia (*p* < 0.001; Figure 2), with the prevalence of moderate myopia being the highest, and the prevalence of high myopia seemed to be decreasing. As for females, the prevalence of moderate myopia was also the highest, but there was no significant change among the prevalence of low myopia, moderate myopia, and high myopia (*p* = 0.081).

### 3.4. Prevalence of myopia in different types of high schools

The prevalence of myopia varied among different types of high schools, with an overall myopia prevalence of 79.6, 85.2, and 86.1% in vocational high schools, general high schools, and key high schools, respectively (*p* < 0.001). *Post hoc* tests showed that the prevalence of myopia in vocational high school was significantly lower than that in the other two types of high schools, while there was no significant difference between general and key high schools.

**Figure 3:**
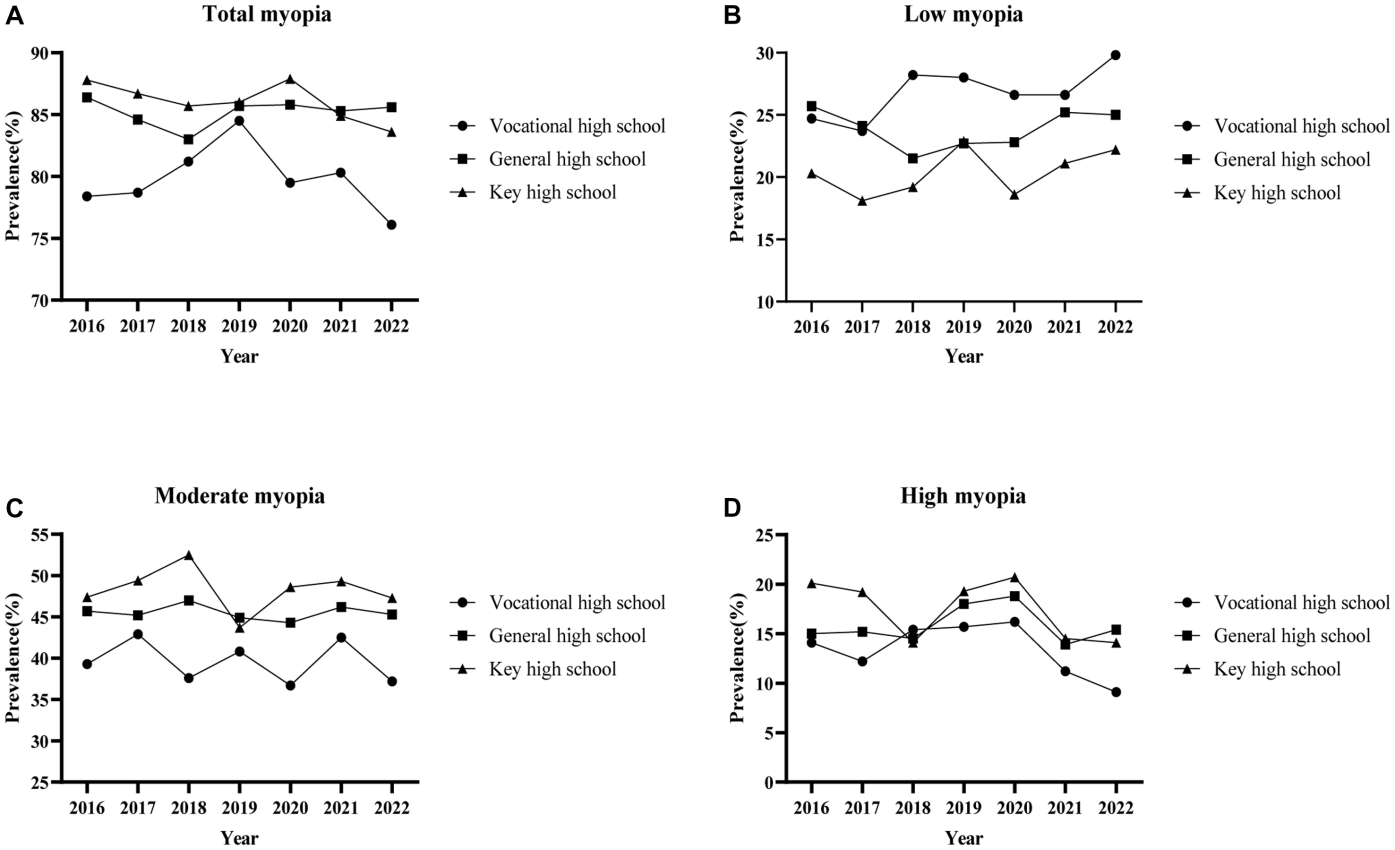
Trends in prevalence of different degrees of myopia by high school. **(A)** Total myopia; **(B)** Low myopia; **(C)** Moderate myopia; **(D)** High myopia.

Table 4 and Figure 3 showed the prevalence of total myopia, low myopia and moderate myopia among senior students in all three types of high schools basically stayed stable (total myopia: vocational high school *p* = 0.087, general high school *p* = 0.087, key high school *p* = 0.492; low myopia: vocational high school *p* = 0.527, key high school *p* = 0.455; moderate myopia: vocational high school *p* = 0.419, general high school *p* = 0.794, key high school *p* = 0.255, except for low myopia in general high school *p* = 0.036). Meanwhile, the prevalence of high myopia in general and key high schools seemed to be fluctuating (general high school *p* = 0.001, key high school *p* = 0.007), with a slightly downward trend in vocational high schools (*p* = 0.013).

**Table 4:** The prevalence of myopia in different types of high school in Fenghua, from 2016 to 2022.

Year	Vocational high school (%)				General high school (%)				Key high school (%)			
	Total myopia	Low myopia	Moderate myopia	High myopia	Total myopia	Low myopia	Moderate myopia	High myopia	Total myopia	Low myopia	Moderate myopia	High myopia
2016	78.4	24.7	39.3	14.4	86.4	25.7	45.7	15.0	87.8**	20.3	47.4	20.1
2017	78.8	23.7	42.9	12.2	84.5	24.1	45.2	15.2	86.7*	18.1	49.4	19.2
2018	81.2	28.2	37.6	15.4	83.0	21.5	47.0	14.5	85.8	19.2	52.5	14.1
2019	84.5	28.0	40.8	15.7	85.6	22.7	44.9	18.0	86.0	22.9	43.7	19.4
2020	79.5	26.6	36.7	16.2	85.9	22.8	44.3	18.8	87.9*	18.6	48.6	20.7
2021	80.3	26.6	42.5	11.2	85.3	25.2	46.2	13.9	84.9*	21.1	49.3	14.5
2022	76.1	29.8	37.2	9.1	85.7	25.0	45.3	15.4	83.6**	22.2	47.3	14.1
Total	79.6	27.0	39.5	13.1	85.2	23.9	45.5	15.8	86.1**	20.3	48.3	17.5
*P* value	0.087	0.527	0.419	0.013	0.087	0.036	0.794	0.001	0.492	0.455	0.255	0.007

Chi-square test, *p < 0.05, **p < 0.001.

### 3.5. Changes in the prevalence of myopia before and during the COVID-19 pandemic

The COVID-19 pandemic broke out at the end of 2019, so we defined the time interval as “before the COVID-19 pandemic (from 2016 to 2019)” and “during the COVID-19 pandemic (from 2020 to 2022).” Table 5 showed that there’s no significant change in the prevalence of overall myopia (84.7% *vs* 84.3%, *p* = 0.425), low myopia (23.3% *vs* 24.3%, *p* = 0.127), moderate myopia (45.5% *vs* 44.7%, *p* = 0.279), and high myopia (15.9% *vs* 15.3%, *p* = 0.274) before and during the COVID-19 pandemic. Stratified analysis based on gender (male *p* = 0.207; female *p* = 0.918) and different school type (vocational high school *p* = 0.112; general high school *p* = 0.299; key high school *p* = 0.393) also confirmed that there was no remarkable change in the prevalence of myopia before and during COVID-19.

**Table 5:** Prevalence of myopia before and during the COVID-19 pandemic.

Parameters	Myopia prevalence(%)		
	Before COVID-19 (2016–2019)	During COVID-19 (2020–2022)	*P* value^a^
Total myopia	84.7	84.3	0.425
Non myopia	15.3	15.8	
Low myopia	23.3	24.3	0.127
Moderate myopia	45.5	44.7	0.279
High myopia	15.9	15.3	0.274
Gender			
Male	82.2	81.1	0.207
Female	86.9	87.0	0.918
Type of high school			
Vocational high school	80.9	78.4	0.112
General high school	84.9	85.6	0.299
Key high school	86.6	85.5	0.393

COVID-19, Corona Virus Disease 2019; ^a^Chi-square test.

## 4. Discussion

Existing studies generally indicate that Asia, especially East Asia, has a high prevalence of myopia (5, 6, 8, 15). A large retrospective review found that 73% of school children in East Asia have myopia, compared with approximately 40% of Europeans and less than 10% of children in African and South American (15). The prevalence of myopia among high school students aged 15 to 19 in Singapore was 73.9% (1), the age-standardized prevalence of myopia among children aged 12 to 18 in South Korea was approximately 80% (15), and our study also showed a high myopia prevalence of 84.5% among senior students in Fenghua City. In contrast, in Germany, only 23% of boys and 35% of girls aged between 14 and 17 years old were diagnosed with myopia (16). On the one hand, ethnic group plays a role in myopia severity. It was reported that myopia progressed faster in Asian American children than in Hispanic, black, and Native American children (17). On the other hand, educational pressure also matters (18–21). It is believed that the prevalent after-school tutoring and intense education began at a young age in school children promote the high prevalence of myopia in Asia (22, 23).

Our study was conducted in Fenghua city, which is located in the eastern coastal region of China, with a regional GDP of over 1,600 billion and a *per capita* GDP of about 137,000 yuan (24). It is generally known that eastern China has a much higher level of economic development and educational attainment than the majority of the rest of the country. We compared the prevalence of myopia in Fenghua City with other similar age groups of adolescents in China (Table 6). The prevalence of myopia among students in eastern cities is generally higher, with two cities in Jiangsu Province reporting prevalence rates of 86.8% for high school students (11, 30). The prevalence of myopia among adolescents in some northern cities was even above 90%, with the prevalence of high myopia reaching more than 20% (32, 33, 36, 37). In contrast, myopia prevalence is relatively low in some western and minority regions such as Xinjiang, with 80.5% of adolescents aged 14 to 18 (38). Overall, myopia prevalence among senior students in Fenghua is above the national average, which may be related to the more developed economy and higher educational pressure in the eastern region.

**Table 6:** Studies of myopia and high myopia prevalence among adolescents in China during the past decade.

Author	Publication year	Province	*N*	Age group/Grade	Prevalence of myopia	Prevalence of high myopia
Lv et al. (25)	2012	Shandong	2,053	18.3 ± 1.8	84.1^a^	/
You et al. (26)	2014	Beijing	1,278	18	72.8^e^	9.1^*^
Wu et al. (27)	2015	Beijing	3,773	16–18	80.7^e^	9.9^*^
Song et al. (28)	2017	/	53,010	16–18	83.3^a^	/
Wei et al. (29)	2018	Henan	1,469	18	83.4^a^	12.1^*^
Chen et al. (10)	2018	Zhejiang	2,932	18.3 ± 0.6	87.7^a^	17.5^*^
Huang et al. (30)	2019	Jiangsu	968	19.6 ± 0.9	86.8^c^	/
Jiang et al. (31)	2020	Zhejiang	16,309	Senior high school	79.2^a^	/
Wang et al. (9)	2020	Zhejiang	403	Grade 12	89.8^d^	26.1^**^
Bai et al. (32)	2022	Tianjin	526	18.34	92.4^a^	20.9^*^
Zhang et al. (33)	2022	Shandong	50,939	Grade 12	94.9^b^	25.1^*^
Chen et al. (11)	2022	Jiangsu	8,267	Senior high school	88.4^a^	/
Yang et al. (7)	2022	Shanxi	2,338	16–18	86.8^a^	/
Zhao et al. (34)	2022	Shanxi	4,874	15–19	90.6^a^	12.4^*^
Wang et al. (35)	2022	Chongqing	294	Grade 11	88.7^a^	/
		Tibet	167		74.4^a^	

Definition of myopia: ^a^UCVA < 5.0 or/and SE < −0.5D; ^b^SE < −0.75D; ^c^Self-reported; ^d^UCVA < 20/25 and SE < −0.5D; ^e^SE ≤ −1.0D.

Definition of high myopia: ^*^SE ≤ −6.0D; ^**^UCVA < 20/25 and SE ≤ −6.0D.

Also, we compared the results with our previous study which analyzed the myopia prevalence among senior students in Fenghua from 2001 to 2015. The prevalence of myopia in Fenghua was 87.7% in 2015, with a prevalence of myopia of up to 90.8% in females, and a prevalence of high myopia of 16.6%. It seems that there’s been a slightly downward trend in myopia prevalence recently. In last several years, the Chinese government attached great importance to the vision health of adolescents, promulgating a series of guidelines on myopia prevention and control. In 2018, the Ministry of Education and the National Health Commission jointly issued an implementation plan for myopia prevention and control among children and adolescents, raising myopia prevention and control to a national strategy (39), the core of which is increasing outdoor activities and reducing educational pressure (40). It is stated that by 2023, efforts should be made to achieve an annual reduction of more than 0.5% in the overall myopia prevalence rate among children and adolescents nationwide from the 2018 baseline. In the current study, the prevalence of myopia among senior students in Fenghua City shows a decreasing trend since 2019, and the annual decrease in myopia prevalence from 2019 to 2022 is 0.4, 0.9, and 1.0% respectively, which have basically achieved the expected target. Meanwhile, we found that the prevalence of high myopia among senior students has also shown a slightly decreasing trend in recent years, which seems to be a good phenomenon. In terms of gender, we found that the prevalence of myopia among female students was higher than that of male students, which was consistent with previous studies (7, 16, 17).

As mentioned above, educational pressure has been recognized as one of the risks factors for myopia (18–21), so we did further analysis by stratifying students by high school. Students in key high schools typically have heavier study tasks, with more educational pressure and less outdoor activity (34). By contrast, students in general high schools have less homework and less educational pressure. The vocational high schools, however, focus on cultivating students’ occupational skills. It includes a large number of practice courses in the curriculum, and students do not have to spend long hours burying in books. As expected, our study found that the prevalence of myopia among senior students in vocational high schools was significantly lower than that in general and key high schools, which may be benefit from the abundant practical courses and relatively mild educational pressure in vocational high schools. High level of educational pressure comes along with long hours of homework, less outdoor times, and continuing near work, all of which play roles in myopia onset and progression (15, 41, 42). Interestingly, we found no significant difference between general and key high schools. One possible reason is that students in both types of schools need to take the college entrance examination, and one of their major tasks is to get higher scores in it, which may lead to a similar load of educational stress. Also, this kind of exam-oriented teaching mode makes students spend most of their study time in textbooks, no matter it’s in key high schools or general high schools.

We also found there’s no significant effect of the COVID -19 pandemic on the prevalence of myopia among senior students in Fenghua City. After the novel coronavirus outbreak in late 2019, the Chinese government implemented a strict home quarantine policy. Schools were shut down nationwide at the end of January 2020 (43), and online courses substituted offline classes. Many surveys have shown a significant reduction in outdoor exercise time, increased electronic devices use, and longer near-work hours for students during home quarantine (44). For example, a study conducted in Shanghai reported that children spent 5.24 h per day on digital devices during home quarantine on average, compared to only around 0.67 h before (44). Similarly, another research reported a reduction of 0.8 h per day in outdoor time and an increase of 4.1 h per day in screen time (13). However, researchers have reached less consistent conclusions regarding the effect of the COVID -19 pandemic on the prevalence of myopia in children and adolescents. Here are some hypotheses that might explain the differences between studies.

First of all, it seems that age may be a significant contributor to this difference. It’s worth noting that studies reporting statistically significant differences in increased prevalence of myopia and more negative spherical equivalent after the epidemic outbreak were most likely conducted on younger children (12–14, 45). In contrast, other studies covering a wider group of age showed that the increase in myopia prevalence during the COVID -19 pandemic was less in older children than in elementary school students (14), and some studies even found little change in myopia prevalence in upper-grade adolescents (12, 46), which is consistent with our findings. Previous studies have repeatedly confirmed the relation between age and myopia progression, with the onset and rapid progression of myopia most often occurring during the elementary school years (17, 37), and the development of myopia slows down after puberty (3, 17, 37, 47, 48). As the axial length has generally stabilized (49), the refractive state of senior students included in our study may no longer be sensitive to environmental changes.

Another possible reason is that the environment changes brought by home quarantine may not yet reach the threshold where they can have an impact on myopia progression (44). Influenced by China’s college entrance examination system, high school students have a strong educational workload, and they rarely engaged in outdoor activities even before the COVID -19 pandemic. At the same time, with the popularity of multimedia teaching, more and more schools are using electronic screen-casting devices or even tablet PC for auxiliary teaching, and students have long been exposed to various electronic devices in daily lives. Therefore, the impact of the online teaching model may not be as significant as we thought.

Additionally, though current studies generally agree that outdoor activity is a protective factor for myopia onset (6, 11, 15, 16, 41), researchers have also shown that its effect on myopia progression does not seem to be significant in those who already have myopia (17, 44, 50). Such differences in the effect of outdoor activities on people with and without myopia may be another reason for the lack of statistical significance in myopia prevalence after the COVID-19 pandemic in our study. In addition, after the outbreak, the Chinese government attached great importance to the vision health of adolescents and issued a series of guidelines and requirements on myopia prevention, refractive screening, and optometry health protection during the online study, which also played a positive role in controlling the development of myopia.

Our study has several limitations. The refractive data collected for this study were from noncycloplegic autorefraction, because there are difficulties of using cycloplegic in large population health screening, and it is challenging to implement in practice. However, it has been confirmed that there is a high agreement between cycloplegic refraction and noncycloplegic optometry results (51), the latter of which is adequate and has been recommended for screening in populations such as schools (52, 53). Another limitation is the lack of detailed information about reading study time, outdoor activity time, time for extracurricular practical activities and time spent on online courses and digital devices among students in different type of schools before and during the COVID-19 pandemic. Thirdly, we lack the assessment of risk factors associated with myopia, such as axial length, parental myopia, genetic factor, socioeconomic characteristics, and writing posture, mainly because this is a large retrospective study and it is difficult to complete additional questionnaire collection. However, this study provides valuable information for myopia prevention and control among Chinese adolescents.

## 5. Conclusion

In conclusion, the prevalence of myopia among senior students in Fenghua was still high but remained stable from 2016 to 2022. Students in vocational high school showed a lower myopia prevalence than students in general high school and key high school. COVID-19 pandemic did not affect the prevalence of myopia among students of this age group. Further attention and more efforts should be paid to myopia prevention and control in the future.

## Data availability statement

The original contributions presented in the study are included in the article/Supplementary material, further inquiries can be directed to the corresponding authors.

## Ethics statement

The studies involving human participants were reviewed and approved by the ethics committee of Fenghua People’s Hospital. Written informed consent from the participants’ legal guardian/next of kin was not required to participate in this study in accordance with the national legislation and the institutional requirements.

## Author contributions

XZ, TL, and KW designed research. BC and AW conducted research. MC and AW analyzed data. XZ and BC performed statistical analysis. TL and KW wrote the draft of the manuscript. MC revised the manuscript. All authors read, reviewed and approved the final manuscript.

## Conflict of interest

The authors declare that the research was conducted in the absence of any commercial or financial relationships that could be construed as a potential conflict of interest.

## Publisher’s note

All claims expressed in this article are solely those of the authors and do not necessarily represent those of their affiliated organizations, or those of the publisher, the editors and the reviewers. Any product that may be evaluated in this article, or claim that may be made by its manufacturer, is not guaranteed or endorsed by the publisher.
